# No hybrid snowcocks in the Altai—Hyper‐variable markers can be problematic for phylogenetic inference

**DOI:** 10.1002/ece3.8199

**Published:** 2021-10-05

**Authors:** Martin Päckert

**Affiliations:** ^1^ Senckenberg Natural History Collections Museum of Zoology Dresden Germany

## Abstract

A recent article in Ecology and Evolution featured the discovery of hybrid snowcocks (*Tetraogallus*) and speculated on the hybrid origin of an extant species (*T. altaicus*). Comprehensive re‐analyses of original data from the latter paper reliably refute the phylogenetic hypothesis taken as firm evidence of a past hybridization event in these birds. The new re‐analyses showed that there is no evidence of hybridization in these snowcocks from the data available so far.

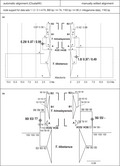

## 1 INTRODUCTION

According to recent genomic studies, speciation with gene flow seems to be more frequent than previously believed (Feder et al., 2012; Nosil, 2008) and hybridization was suggested to play a so‐far underestimated role in speciation processes (Abbott et al., 2013; Ottenburghs, 2018). Whole‐genome data have detected traces of past gene flow between ancestors of extant species in a couple of vertebrate examples (Jónsson et al., 2014; Thom et al., 2018). However, hybrid origin of an extant species still seems to be rare in terrestrial vertebrates, for example, for birds, a review paper by Ottenburghs (2018) lists only seven hybrid species proposed to date. Since then, recent studies have added a few further candidate species of possible hybrid origin to this rather short list, such as Salvin's prion, *Pachyptila salvini* (Masello et al., 2019), and Steller's eider, *Polysticta stelleri* (Lavretsky et al., 2021). Several bird species were shown to hybridize with more than one congener (Ottenburghs, 2019), and as a very rare outcome of such crossings, some hybrid individuals were suggested to have derived from successive interspecific and intrageneric hybridization (thus having an admixed hybrid genome of three species; Toews et al., 2018).

In a recent paper on the diversification of *Tetraogallus* snowcocks (Galliformes, Phasianidae), Ding et al. (2020) reported on the discovery of three putative hybrid individuals that they supposed to have originated from a hybridization event between the Himalayan snowcock (*T*. *himalayensis*) and the Tibetan snowcock (*T*. *tibetanus*). These three birds had originally been identified as *T*. *himalayensis* by Wang et al. (2011) and carried to two distinct D‐loop haplotypes (H35, H36). From Wang et al. (2011), there is no information on a deposition of any specimen that would refer to these two haplotypes, so for the time being we must assume that Wang et al. (2011) at best had seen the birds and identified them as *T*. *himalayensis* based on their phenotype. However, in Ding et al.’s (2020) re‐analysis of the D‐loop data set by Wang et al. (2011), the two crucial *T*. *himalayensis* sequences (H35 and H36) clustered with *T*. *tibetanus*. Ding et al. (2020) took this as firm evidence of having detected previously undocumented hybrid specimens that must have originated from a putative recent event when “the male *T*. *himalayensis* hybridized with the female *T*. *tibetanus*.” To be precise, at this stage of analysis, Ding et al. (2020) were discussing two snowcock specimens that someone else had identified as Himalayan snowcocks, *T*. *himalayensis*, and that contrary to expectations, they believed to carry a mitochondrial haplotype of another species‐level lineage, that is, that of the Tibetan snowcock, *T*. *tibetanus*. This alone is not indicative of hybrid origin of these specimens, because there are other obvious explanations for this supposed mismatch between taxon assignment at GenBank and molecular species identification, for example a simple misidentification (Tritsch et al., 2017). The result might also be indicative of mitochondrial introgression into a phenotypical *T*. *himalayensis* population with a nuclear gene pool of that species. Readers would therefore expect further and stronger evidence from molecular data for the hybrid hypothesis. However, instead Ding et al. (2020) took the unexpected position of the *T*. *himalayaensis* specimens in the D‐loop tree as firm evidence of a putative hybrid population existing in the area of sympatry of the two putative parental species. The origin of that theoretical hybrid population (*T*. *himalayensis* × *T*. *tibetanus*) was dated back to the Early Pleistocene at about 1.83 Mya. Furthermore, (Ding et al., 2020) hypothesized that the extant Altai snowcock, *T*. *altaicus*, should have emerged from that postulated hybrid population. Thus, according to Ding et al. (2020), the Altai snowcock should be the next candidate to be added to the shortlist of avian hybrid species. They put this hypothesis to test using a multi‐locus data set of five mitochondrial markers and four nuclear markers for six species (four *Tetraogallus* sp. and two *Alectoris* sp.) each of them represented by a single concatenated sequence.

However, a sampling that includes a single individual of a hypothesized hybrid taxon and a single individual of each of the putative parental taxa has barely any explanatory power. Moreover, a closer look at the results presented by Ding et al. (2020) reveals further peculiarities and striking deviations from expectations on their postulated hybrid scenario.

As concerns the postulated hybrid origin of the Altai snowcock, their argumentation suffers from a striking inconsistency: If that putative hybrid form *T*. *altaicus* indeed would have originated from the past hybridization event assumed by Ding et al. (2020), it should carry the same mitochondrial lineage as the putative hybrids H35/H36 (or a haplotype derived from that lineage). Considering the ratio of mtDNA and nuclear markers of 5: 4 in the multi‐locus data set, we should expect a strong mitochondrial signal and thus a closer relationship of the putative hybrid species *T*. *altaicus* to the postulated female parent, that is, *T*. *tibetanus*. However, the opposite is the case: The multi‐locus phylogeny by Ding et al. (2020) showed a sister‐group relationship of *T*. *altaicus* and the presumed male parent, *T*. *himalayensis* (Ding et al., 2020: figure 2). Taking into account that the hybrid origin of *T*. *altaicus* under the scenario developed by Ding et al. (2020) would be realistic, extremely strong mito‐nuclear discordance could be one (if not the sole) alternative explanation for this unexpected topology (Bonnet et al., 2017; Toews & Brelsford, 2012).

Identification of hybrid individuals using a single mitochondrial marker (D‐loop as in case of haplotypes H35/H36) seems even less convincing. However, if we acknowledged the hybrid origin of these specimens from cross‐breeding between a female *T*. *tibetanus* and a male *T*. *himalayensis*, then we would expect the hybrid offspring to carry any of the known recent haplotypes of the female parental species or at least a haplotype that was firmly nested in one of the parental clades. The Italian sparrow, *Passer italiae*, that was reliably shown to have originated from past hybridization between two sparrow species (*P*. *domesticus*, *P*. *hispaniolensis*) during the late Pleistocene is a perfect example: In all populations genotyped so far, haplotypes of the house sparrow (*P*. *domesticus*) lineage occur with frequencies near 100% (Hermansen et al., 2011, 2014; Päckert et al., 2019). However, the situation in *Tetraogallus* snowcocks is different: The two putative hybrids haplotypes H35 and H36 were not “embedded in the *T*. *tibetanus* clade” as, claimed by Ding et al. (2020), they were sister to this clade separated by a deep split dated at 1.83 Ma from all *T*. *tibetanus* (figure 5 in Ding et al., 2020).

Finally, a cross‐check of the original paper by Wang et al. (2011; who generated the D‐loop data set and analyzed it before) reveals one striking deviation from the D‐loop tree by Ding et al. (2020): In the D‐loop phylogeny by Wang et al. (2011), the two sequences H35 and H36 (originally inferred from three *T*. *himalayaensis* specimens) belonged to the *T*. *himalayensis* clade with full support. Thus, H35/H36 could also represent descendants of an ancient mitochondrial lineage of the Himalayan snowcock. However, the conflicting position of the latter two haplotypes (H35, H36) in the two previous studies could be indeed due to deficient sampling, because Wang et al. (2011) used only two *T*. *tibetanus* sequences to root their D‐loop phylogeny. Thus, a more comprehensive sampling of that latter species in Ding et al.’s (2020) study plus a different choice of more distantly related outgroups (*Alectoris*) might explain the different topologies with respect to the position of haplotypes H35 and H36.

All these striking peculiarities and contradictions in Ding et al.’s (2020) study require further explanation; however, they have not been put to test yet. Therefore, I decided to re‐examine their entire molecular data sets to evaluate reasons for (i) the mismatch of the position of haplotypes H35 and H36 in the D‐loop trees by Ding et al. (2020) and by Wang et al. (2011), and (ii) the unexpected sister‐group relationship of the putative hybrid species *T*. *altaicus* and the postulated male parent *T*. *himalayensis* in the multi‐locus tree by Ding et al. (2020).

## 2 METHODS

### 2.1 Mitochondrial markers

To control for the position of the three putative hybrid specimens (haplotypes H35, H36) in the *Tetraogallus* tree, I downloaded the original D‐loop sequences used by Ding et al. (2020) from GenBank. Because there was no information on alignment procedure in Ding et al. (2020), I applied the ClustalW algorithm (Higgins et al., 1994) in MEGAX (Kumar et al., 2018). From that first alignment, it was evident that original GenBank sequences had different lengths, because they were gathered from two different studies: All *T*. *himalayensis* were 1154–1155 bp long (Wang et al., 2011: GenBank accession numbers GQ343513–GQ343549), whereas most sequences of *T*. *tibetanus* were shorter and comprised only 883–884 bp of the D‐loop (An et al., 2015: GenBank accession numbers JX136799–JX136833). Ding et al. (2020) did not provide information whether they cut down the alignment to the same sequence length or whether they used a full‐length alignment allowing for missing data in *T*. *tibetanus*. Therefore, I decided to use both the full‐length and the cut‐down sequence data set for phylogenetic reconstructions.

The D‐loop sequence set used by Ding et al. (2020) used for re‐analysis contained 74 sequences: *T*. *himalayensis* haplotypes H1–37, *T*. *tibetanus* haplotypes H1–H35, and two outgroup sequences (*Alectoris chukar*, *A*. *rufa*; compare Table S1). For data set 1, those 74 sequences were cut down to 890 bp, whereas data set 2 contained full‐length sequences in an alignment of 1163 bp with missing data for the last 273 bp of most *T*. *tibetanus* sequences.

A closer look revealed that the putative hybrid specimens (haplotypes H35, H36) differed greatly from all other sequences only in a short hyper‐variable region of the D‐loop (about 100 bp long). In the cut‐down alignment (890 bp), ClustalW incorporated a deletion (gap) at position 119 in sequences H35 and H36. However, if this gap is deleted and the deletion is shifted to position 139 in the 890‐bp alignment, D‐loop sequences of H35 and H36 look surprisingly similar to *T*. *tibetanus* sequences, whereas in other parts of the sequence nearly all variable sites are shared between H35, H36, and *T*. *himalayensis*. Thus, the sister‐group relationship of H35/H36 and *T*. *tibetanus* might be strongly influenced by the position of a single deletion in the hypervariable region of the D‐loop. To see whether the inclusion of further sequence information for the more conservative second fragment had an effect on tree topology, I downloaded further full‐length sequences of the 1163‐bp D‐loop fragment of *T*. *tibetanus* and *T*. *himalayensis* from whole mitochondrial genomes from GenBank (KY766921, NC_023939, KF027439; GQ343550, GQ343551; KY766922) and included these in another alternative data set 3 (= data set 2 + the latter 6 D‐loop fragments from whole mitogenomes; 1163 bp, *n* = 80 sequences).

Because manual editing of automatically aligned sequences is a common procedure, I took into account that Ding et al. (2020) might have re‐edited their alignment manually. Therefore, I aligned each of the three data sets under two different strategies: (1) automatic alignment by ClustalW (under default settings; deletion at position 119 of the 890‐bp alignment) and (2) ClustalW alignment with manual editing of H35 and H36 for similarity of the hypervariable region with *T*. *tibetanus* (deletion at position 139; see above).

Strikingly, Ding et al. (2020) did not include a D‐loop sequence of the putative hybrid species *T*. *altaicus* neither in the D‐loop data set nor in the multi‐locus data set, presumably because of a lack of sequence data for this species at that time. However, meanwhile a full mitochondrial genome of the Altai snowcock, *T*. *altaicus*, was published by Kimball et al. (2021). If Ding et al.’s (2020) hypothesis on the hybrid origin of *T*. *altaicus* from an admixed ancestral population carrying the putative ancestral hybrid lineage of haplotypes H35/H36 was reliable, then the D‐loop sequence of *T*. *altaicus* should be closely related to the latter two haplotypes. Furthermore, a clade uniting H35/H36 and *T*. *altaicus* should be sister to (or embedded in) a clade of the putative female parent *T*. *tibetanus* (compare Ding et al., 2020). To verify their hypothesis on the phylogenetic relationships of *T*. *altaicus*, I repeated single‐locus and multi‐locus analyses with the D‐loop sequence of *T*. *altaicus* included.

To check whether other mtDNA markers would support a similar branching pattern like the D‐loop, I also used a cytochrome *b* data set (from Ruan et al., 2010) for comparison of intra‐ and interspecific diversification (Table S2).

### 2.2 Multi‐locus sequence data

To control for the position of the putative hybrid form *T*. *altaicus* in the *Tetraogallus* tree, I downloaded original sequences included in the multi‐locus‐data set of 9 loci by Ding et al. (2020) from GenBank (Table S3). During multi‐locus alignment preparation, I realized that ND2 sequences of the two outgroup species *Alectoris rufa* and *A*. *chukar* (DQ307002, FJ752426) were nearly identical. This appeared rather unreliable, because the two coding mtDNA markers (COI and cytb) differed greatly among the two species and so did the non‐coding mtDNA markers (D‐loop, 12S rRNA). Because Ding et al. (2020) used the split among these two outgroup species for time calibration of their multi‐locus phylogeny, I assumed a notable effect of that mismatch among mtDNA markers on divergence times. Indeed, divergence time estimates by Ding et al. (2020: figures 2 and 4)) inferred from the multi‐locus tree are nearly twice as old as those inferred from their time‐calibrated D‐loop tree. No plausible explanation for such divergence was provided by Ding et al. (2020); however, we might assume that this could be due to the use of an inappropriate ND2 sequence for one of the outgroups. Therefore, I downloaded a comprehensive data set of ND2 sequences for *Alectoris* and *Tetraogallus* to control for the position of the two partridge outgroup sequences DQ307002, FJ752426 (Table S4).

### 2.3 Inference of phylogeny

For Bayesian inference of phylogeny using BEAST v.1.8.1 (Drummond et al., 2012), I applied exactly the same settings as provided in Ding et al. (2020): MCMC chain length of 50 Mio generations, sampling every 1000th generation, and a burn‐in of 10% applied. I also referred to the same time calibration as Ding et al. (2020) who assumed a fixed node age for the time of the most recent common ancestor (tmrca) of the two partridge species used as outgroups: 2.84 Ma for the split between *Alectoris chukar* and *A*. *rufa*, mean tmrca prior starting value, *SD* = 0.01 (compare Ding et al., 2020). Following Ding et al. (2020), I applied the HKY + I + G to the D‐loop data set. Though Ding et al. (2020) claimed that they had a priori identified “best partition schemes for the dataset,” they applied a single model (GTR + I) across all 9 loci of the multi‐locus data set. Because they stated that they had used concatenated sequence data for Bayesian inference of phylogeny with BEAST, I must assume that they treated their entire multi‐locus alignment as a single partition. Although there are more appropriate alternatives for partitioning of a multi‐locus data set including coding and non‐coding mtDNA and nuclear markers, I decided to stick closely to Ding et al.’s (2020) protocols and treated the whole multi‐locus data set as one partition.

Moreover, Ding et al.’s (2020) hypothesis of a hybrid origin of *T*. *altaicus* was based on the assumption that the putative hybrids must carry haplotypes of the mitochondrial lineage of *T*. *tibetanus* (because they were supposed to have emerged from hybridization between a male *T*. *himalayensis* and a female *T*. *tibetanus*). Strikingly, *T*. *altaicus* was sister to *T*. *himalayensis* in the multi‐locus tree by Ding et al. (2020) and not to *T*. *tibetanus*. If there was any support for Ding et al.’s (2020) theory of a hybrid origin of *T*. *altaicus*, then this tree topology can only have arisen from an extremely strong signal of the nuclear markers that would have masked the mitochondrial signal (sister‐group relationship with *T*. *altaicus* and the putative female parent species *T*. *tibetanus*). This can be doubted, because together the nuclear markers comprised only about half as many base pairs as the mitochondrial markers, thus nuclear markers made up only about one third of Ding et al.’s (2020) concatenated alignment. To control for such an effect, I performed phylogenetic reconstructions for separate data sets of mtDNA (COI, cytb, ND2, 12S rRNA, and D‐loop) and nuclear markers (CLTC, CLTC1, EEF2, RHO; compare Table A1 in Ding et al., 2020). I had to remove *T*. *caspicus* from the nuclear data set, because none of the nuclear markers was available for that species. When according to the model settings in Ding et al. (2020) the GTR + I model was applied to the nuclear data set, BEAST runs were always aborted due to a numerical likelihood error, no matter which prior settings were modified. I had to apply the simpler HKY + I + G model (that Ding et al., 2020 had selected for single‐locus analysis of the D‐loop data set) and include *Francolinus swainsonii* as a further outgroup in accordance with the tree topology in Wang et al. (2013). Then, BEAST ran smoothly for 50 Mio generations for the set of four concatenated nuclear loci.

To check for adequate ESS values for all parameters, I used TRACER v. 1.4 (Rambaut & Drummond, 2007).

Apart from Bayesian inference of phylogeny with BEAST, Ding et al. (2020) did not consider any alternative reconstruction methods. As a control, I used a maximum‐likelihood approach using RaxML (Stamatakis, 2006, 2014) with the GTR + I + Г model applied for tree reconstruction with all data sets and alignments. All obtained phylograms were edited in FIGTREE vers. 1.4.2 (Rambaut, 2009).

For illustration of mitochondrial lineage differentiation, I reconstructed unrooted minimum spanning networks with PopART (http://popart.otago.ac.nz) and with TCS (Clement et al., 2000) to check for a potential effect of gaps in the D‐loop alignment. For calculation of uncorrected pairwise distances between species and intraspecific mitochondrial lineages, I used MEGAX (Kumar et al., 2018).

## 3 RESULTS

### 3.1 Mitochondrial markers

**FIGURE 1:**
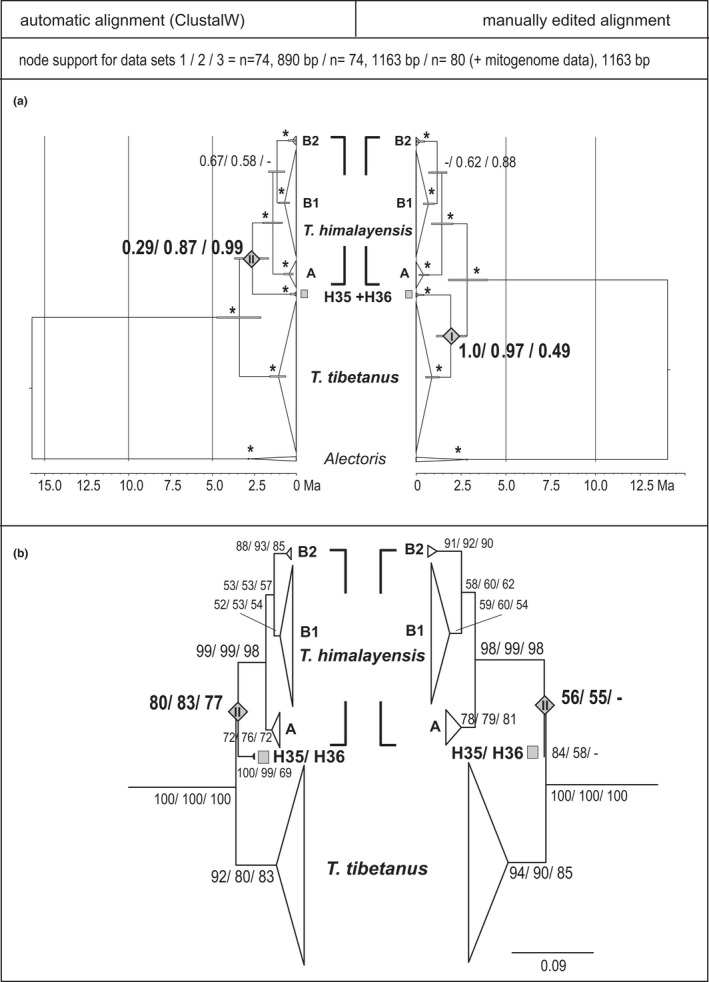
Phylogenetic relationships of enigmatic haplotypes H35 and H36 inferred from three different data sets of mitochondrial D‐loop sequences (1= 890 bp, *n* = 74; 2= 1163 bp, *n* = 74; 3= 1163 bp, *n* = 80) based on two different alignment procedures (automatic alignment by ClustalW (left) and automatic alignment plus manual editing of the hypervariable region of sequences H35 and H36 (right) using two different methods: (a) Bayesian inference of phylogeny; time‐calibrated node *Alectoris* (mean age = 2.84 Ma; below RaxML, node support inferred from 1000 bootstrap replicates); (b) maximum‐likelihood using RaxML; node support inferred from all three data sets indicated as shown in the figure; asterisk indicates full support; diamonds indicate nodes supporting a sister‐group relationship of specimens H35/H36 with either *T*. *tibetanus* (diamond I) or *T*. *himalayensis* (diamond II)

Figure 1 shows a comparison of six independent runs with BEAST (Figure 1a) and RaxML (Figure 1b) using two different alignment options (CLUSTAL W with and without manual editing of sequences H35 and H36) for three data sets: 1 = Ding et al.’s (2020) sampling, *n* = 74, length 890 bp; 2 = Ding et al.’s (2020) sampling, *n* = 74, 1163 bp; 3 = Ding et al.’s (2020) sampling plus further mitogenome data for *T*. *tibetanus* and *T*. *himalayensis*, *n* = 80, length 1163 bp. Only one out of six Bayesian trees matched the D‐loop tree topology shown in Ding et al. (2020) with the same full support for a sister‐group relationship of H35 and H36 with *T*. *tibetanus* (Figure 1, node I): That tree was inferred from the 890‐bp alignment 2 with manual correction for similarity with *T*. *tibetanus* in the hypervariable region. Node support for this relationship decreases in runs with full‐length D‐loop sequences to poor Bayesian support of 0.49 when further full‐length sequences of *T*. *tibetanus* were added to data set 3 in a manually edited alignment (Figure 1, node I). BEAST runs based on the automatic alignment of data sets 1, 2, and 3 did not confirm the sister‐group relationship of H35 and H36 with *T*. *tibetanus*, nor did any of the six RaxML reconstructions (Figure 1, nodes II). All of these showed the same sister‐group relationship of H35 and H36 with *T*. *himalayensis*.

**FIGURE 2:**
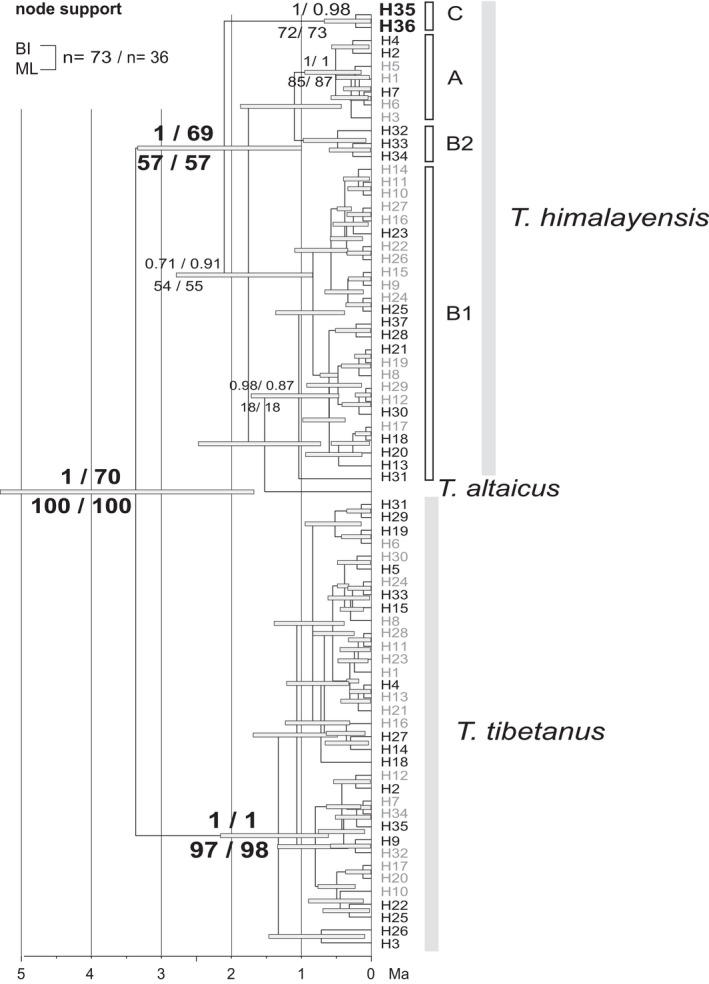
Time‐calibrated phylogeny of *Tetraogallus* snowcocks based on the second conservative fragment of the D‐loop excluding the hypervariable region (751 bp; sequence information for the putative hybrid species, *T*. *altaicus*); node support values from Bayesian posterior probabilities and maximum‐likelihood bootstrap indicated above and below nodes; support values are shown for independent runs with BEAST and RaxML for (1) all samples included (*n* = 75), (2) each distinct haplotype represented by only one sequence (black numbers at tip clades) and duplicate haplotypes deleted (gray numbers at tip clades; total *n*= 38); the *Alectoris* outgroup clade was deleted from the tree

The manually edited 890‐bp alignment of D‐loop sequences contained 80 variable sites of which 63 were parsimony‐informative (the two *Alectoris* outgroup taxa excluded). Among the latter, 26 parsimony‐informative sites were located within the region between site 101 and 138, that is, 41% of the total parsimony‐informative sites were accumulated in a hypervariable region of only 37 bp length. When the entire first fragment including that hypervariable region was cut off, the remaining fragment (751 bp length) contained 37 variable sites (the two *Alectoris* outgroup taxa excluded) of which 28 were parsimony‐informative. At those parsimony‐informative sites, sequences H35 and H36 shared ten (out of 28) substitutions with all *T*. *himalayensis*, whereas only one substitution with *T*. *tibetanus* and another strongly diversified *T*. *himalayensis* haplotype H37. When only that last fragment of the D‐loop (751 bp without the hypervariable region) was analyzed (including sequence information for the putative hybrid form *T*. *altaicus*), H35 and H36 were sister to *T*. *himalayensis* with full support from Bayesian posterior probabilities and moderate support from likelihood bootstrap (Figure 2, Figure S1). Due to removal of the hypervariable region, the number of distinct D‐loop haplotypes decreased from 73 to 36 (*T*. *himalayensis*, *n* = 18; *T*. *tibetanus*, *n* = 15; *T*. *altaicus*, *n* = 1). To avoid inflation of the data set and a possible effect of duplicate identical sequences, the analysis was repeated with the remaining 35 distinct haplotypes. Removal of duplicate haplotypes had no effect on likelihood bootstrap values; however, it evoked a notable decrease of node support from Bayesian posterior probabilities (except for the node uniting clades A, B1, and B2 of *T*. *himalayensis*; Figure 2). Nevertheless, a sister‐group relationship of the supposed hybrid lineage H35/H36 or of the supposed hybrid species *T*. *altaicus* with the putative female parent *T*. *tibetanus* was not supported in any run with BEAST or RaxML based on the second conservative fragment (751 bp; Figure 2, Figure S1). The Altai snowcock, *T*. *altaicus*, was firmly nested in *T*. *himalayensis*; however, its relationships with clades A, B1, and B2 of the latter species were conflicting among phylogenetic reconstructions; for example, in the Bayesian tree *T*. *altaicus* was sister to clade B1 (Figure 2), in the RaxML tree it was nested in clade B2 (not shown).

**FIGURE 3:**
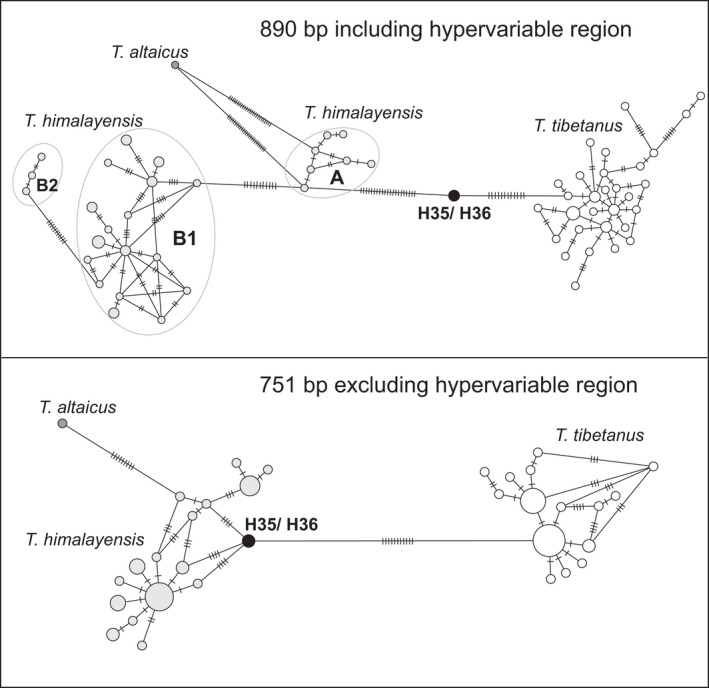
Minimum spanning networks of the D‐loop for data set 1 (890 bp, *n* = 74) based on the manually edited alignment that yielded the same tree topology as shown in Ding et al. (2020); full fragment of 890 bp including the hypervariable region (above); shorter conservative fragment of 751 bp excluding the hypervariable region (below)

Comparison of minimum spanning networks showed that manual editing of D‐loop sequences placed H35 and H36 between haplotype clusters of *T*. *himalayensis* and *T*. *tibetanus* (Figure 3, for data set 1, 890 bp, manually edited alignment: minimum distance of 21 vs. 12 substitutions; 25 vs. 16 substitutions in the TCS network when gaps were treated as a 5th character, Figure S2). Due to the position of H35/H36 closer to *T*. *tibetanus* and to the greater diversification of the *T*. *himalayensis* cluster (groups A, B, C, and *T*. *altaicus* linked to group A; Figure 3), mean uncorrected p‐distances were higher for pairwise comparisons between H35/H36 and *T*. *himalayensis* (2.7% < *p*‐dist < 3.6%) as for comparisons between H35/H36 and *T*. *tibetanus* (*p*‐dist = 1.9%: Table 1, above diagonal). This is a clear effect of the hypervariable region, because removal of the first 139 bp from the D‐loop alignment yielded a much closer relationship of H35 and H36 with the *T*. *himalayensis* cluster (Figure 3: minimum distance H35/H36: *T*. *himalayensis* = 4 substitutions; H35/H36: *T*. *tibetanus* = 11 substitutions; 4 vs. 13 substitutions in the TCS network when gaps were treated as a 5th character, Figure S2). Accordingly, pairwise distances between H35/H36 and the putative female parent *T*. *tibetanus* inferred from the second conservative D‐loop fragment alone were about 2 to 3 times greater (*p*‐dist = 1.6%) than those between H35/H36 and each of the three intraspecific mitochondrial lineages of *T*. *himalayensis* (0.5% < *p*‐dist < 0.9%; Table 1, below diagonal).

**TABLE 1:** Uncorrected genetic distances (p‐dist.) between divergent mitochondrial lineages of the Himalayan snowcock (*Tetraogallus himalayensis*) and close relatives (*T*. *altaicus*, *T*. *caspicus*, and *T*. *tibetanus*); separate calculations for and for the cytochrome *b* data set and the D‐loop data set (below diagonal = 751 bp without the hypervariable region; above diagonal=890 manually edited alignment; missing data for *T*. *caspicus*; pairwise comparison among H35/H36 and the putative female parent *T*. *tibetanus* in bold)

Cytochrome *b*					
	*himalayaensis*	*caspius*	*altaicus*	HIM1	HIM2
*himalayensis*	–				
*altaicus*	0.0167	–			
*caspius*	0.0239	0.0285	–		
HIM1	0.0339	0.0388	0.0452	–	
HIM2	0.0505	0.0593	0.0667	0.0748	–
*tibetanus*	0.0632	0.0645	0.0703	0.0769	0.086

D‐loop						
	HIM A	HIM B1	HIM B2	H35/36	*altaicus*	*tibetanus*
HIM A	–	0.0199	0.0162	0.0274	0.0293	0.0388
HIM B1	0.00882	–	0.0168	0.0309	0.0331	0.0402
HIM B2	0.00528	0.00639	–	0.0321	0.0336	0.0434
H35/36	0.00706	0.00721	0.00623	–	0.0363	**0.0189**
*altaicus*	0.01755	0.01522	0.01335	0.01603	–	0.0449
*tibetanus*	0.02188	0.02098	0.02091	**0.01618**	0.02716	–

Both the D‐loop and the cyt‐*b* data confirmed the existence of several intraspecific splits among mitochondrial lineages of the Himalayan snowcock, *T*. *himalayensis* (Figure 3, Figure S3). In both data sets, mean intraspecific divergence within *T*. *himalayaensis* was greater than mean interspecific divergence between the latter species and *T*. *altaicus* (Figure 3, Figure S3; Table 1). This is another reason why resolution of phylogenetic relationships was problematic for a deeply divergent mitochondrial lineage of *T*. *himalayensis* like H35/H36, particularly when phylogenetic inference was based on a hypervariable marker like the D‐loop.

### 3.2 Multi‐locus data set

**FIGURE 4:**
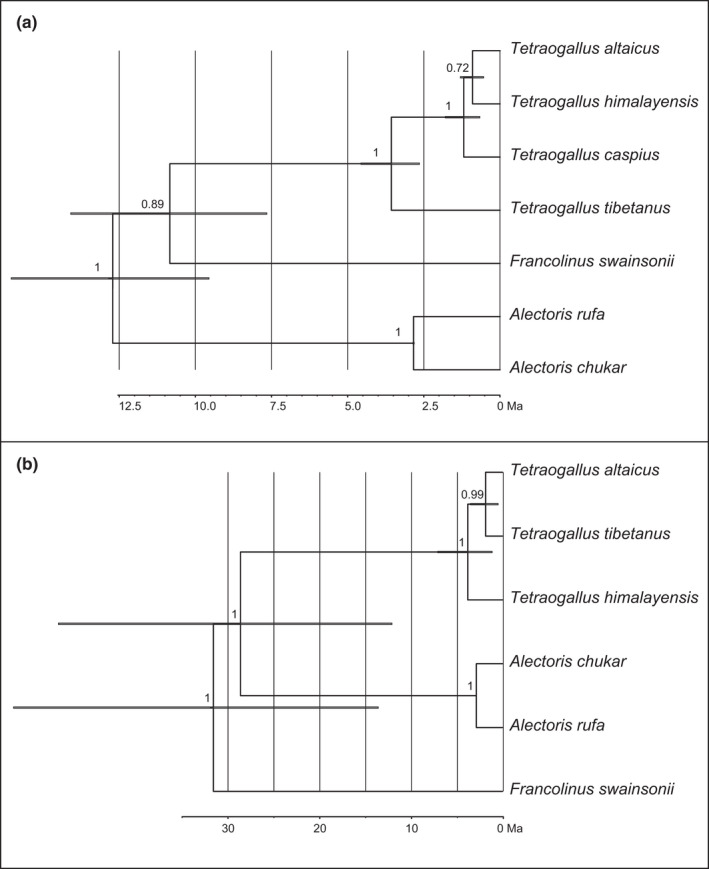
Time‐calibrated phylogenies inferred from the multi‐locus data set by Ding et al. (2020) separately for (a) five mitochondrial markers (COI, cytb, ND2, 12S rRNA, D‐loop), (b) four nuclear loci (CLTC, CLTC1, EEF2, RHO)

Separate tree reconstructions for concatenated mtDNA and nuclear markers showed that *T*. *altaicus* carried a mitochondrial lineage that was closely related to *T*. *himalayensis* (not to the postulated female parent *T*. *tibetanus*), however, with poor support (Figure 4). Both species formed a fully supported monophyletic clade with the Caspian snowcock (*T*. *caspius*; Figure 4). In contrast, the nuclear maker set supported a closer relationship of *T*. *altaicus* with *T*. *tibetanus* (Figure 4). Inclusion of the newly available D‐loop sequence for *T*. *altaicus* (that was missing from the data set used by Ding et al., 2020) did not change neither tree topology nor node support values (not shown). None of the mitochondrial markers analyzed separately confirmed Ding et al.’s (2020) hypothesis on a closer relationship of the Altai snowcock, *T*. *altaicus*, with the postulated female parent, *T*. *tibetanus* (see above).

Comparison of ND2 sequences showed that the *Alectoris rufa* sequence DQ307002 Ding et al. (2020) had selected for multi‐locus analyses was firmly nested in the *A*. *chukar clade* (Figure S4). Thus, their two outgroup species used for time calibration were unnaturally similar in one mtDNA marker, which as a consequence led to unreliably ancient divergence time estimates among snowcock species (Table 2). When the inappropriate outgroup sequence DQ307002 was replaced by another sequence that represented the true *A*. *rufa* lineage, divergence time estimates for all nodes decreased as expected (Table 2).

**TABLE 2:** Divergence time estimates for nodes of the *Tetraogallus* snowcock tree inferred by Ding et al. (2020) from their multi‐locus data set (9 loci) and from the D‐loop data set compared to re‐analysis of Ding et al.’s (2020) original multi‐locus data set including the wrong ND2 sequence for *Alectoris rufa* and an alternative data set including a correct ND2 sequence for *A*. *rufa*; nodes: crown = T. *himalayensis*, *T*. *caspicus*, *T*. *altaicus*; sister = terminal sister species *T*. *himalayensis*, *T*. *altaicus*

Nodes	Ding et al. (2020)		This study			
	9 loci	D‐loop	9 loci, wrong ND2		9 loci, correct ND2	
			Mean	95% HPD	Mean	95% HPD
Root	23.06	12.18	20.51	[8.86–29.36]	16.2	[7.46–22.31]
*Tetraogallus*	5.91	3.54	5.88	[2.48–8.60]	4.62	[2.31–6.79]
Crown	2.28	–	2.16	[0.98–3.48]	1.70	[0.77–2.62]
Sister	1.95	–	1.83	[0.87–2.73]	1.22	[0.73–2.13]

## 4 DISCUSSION

Re‐examination of D‐loop sequence data from Ding et al. (2020; and Wang et al., 2011) reliably showed that the unexpected position of samples H35 and H36 in the *Tetraogallus* tree was strongly affected by alignment procedure and the position of gaps in a hypervariable region (compare Swain, 2018). As a consequence, the sister‐group relationship of H35 and H36 with *T*. *tibetanus*—the sole argument by Ding et al. (2020) for the putative hybrid status of the three specimens carrying those haplotypes—was strongly supported by only one out of six runs with BEAST and by none of the six maximum‐likelihood trees inferred from RaxML analysis.

Although the control region generally appeared to perform equally well for phylogenetic reconstructions as other mitochondrial genes (such as cytochrome *b* for example in Galliformes; Randi et al., 2001), the hypervariable domains I and II can be considerable sources of error in alignments and thus lead to incorrect tree topologies. Particularly the phylogenetic signal of gaps and their position in an alignment can have a strong effect on phylogenies (Dessimoz & Gil, 2010; Lutzoni et al., 2000), even more when shifts of larger sequence fragments are associated with gap positions, like in our snowcock example. Moreover, characteristically strong intraspecific variation of the hypervariable D‐loop can be associated with tandem repeats (such as in bush tits; Wang et al., 2015) which furthermore complicates sequence alignment. In fact, for hypervariable genetic markers alignment strategy can have a considerable effect on tree topologies (e.g., 12S rRNA of birds; Espinosa de los Monteros, 2003). To ensure alignment accuracy, removal of hypervariable regions from alignments prior to phylogenetic reconstruction is therefore a commonly applied strategy, for example, for D‐loop sequences (Robins et al., 2010) or other hypervariable markers like 16S rRNA (Anthes et al., 2008). Removing ambiguously aligned parts from alignments can even make sense for phylogenetic analysis of protein sequence data sets (Talavera & Castresana, 2007). According to these expectations, removal of the first hypervariable D‐loop fragment from the alignment resulted in a fully supported monophyletic *T*. *himalayensis* including sequences H35 and H36.

In fact, Ding et al. (2020) did not discover previously unknown hybrid snowcocks, because haplotypes H35 and H36 just represent another deeply split mitochondrial lineage of the genetically diverse Himalayan snowcock, *T*. *himalayensis* (Figure 1, clades A, B1, B2; compare Wang et al., 2011). Strong intraspecific variation of the Himalayan snowcock was corroborated by two strongly diverged cytochrome *b* lineages of *T*. *himalayensis* (Figure 3) and by a recent microsatellite study (An et al., 2020). Strikingly, the strong intraspecific diversification of *T*. *himalayensis* does not seem to be associated with phylogeographic structure: Although lineage HIM1 was exclusively found in *T*. *h*. *grombczewskii* from the Kunlun Mountains, and *T*. *h*. *koslowi* from Qinghai, several haplotypes from the central *T*. *himalayensis* cluster were found within the range of these two subspecies, too. Range‐wide admixture of distinctive mitochondrial lineages and local sympatry of individuals belonging two different haplogroups was described for other Palearctic birds, such as the common redstart, *Phoenicurus phoenicurus* (Hogner et al., 2012), or the long‐tailed tit, *Aegithalos caudatus* (Zink et al., 2008).

In the end, from the results presented in Ding et al. (2020), there is no evidence of a possible hybrid status of the two specimens H35 and H36 nor is there any evidence of a hybrid origin of the Altai snowcock, *T*. *altaicus*. The lesson learnt from this case study is that results inferred from mitochondrial markers (in particular from those including hypervariable regions) require a thorough quality check (see Botero‐Castro et al., 2016), moreover if specimens are not available and like in the snowcock example any information on taxonomic assignment of DNA sequences is inferred from GenBank information only (compare Hofstetter et al., 2019).

## CONFLICT OF INTEREST

None to declare.

## AUTHOR CONTRIBUTION

**Martin Päckert:** Conceptualization (equal); Formal analysis (equal); Methodology (equal); Validation (equal); Visualization (equal); Writing‐original draft (equal); Writing‐review & editing (equal).

## Supporting information

Fig S1‐S4

Table S1‐S4

## Data Availability

All sequence data used in this study are available at GenBank. For information on sequence data sets accession numbers, please refer to information provided in the Methods and to the full documentation of the sequence data sets in Tables S1–S4.
