# Supplementary material for: No hybrid snowcocks in the Altai—Hyper‐variable markers can be problematic for phylogenetic inference
Source: Ecol Evol. 2021 Oct 5;11(22):16354–64. doi: 10.1002/ece3.8199 (PMC8601899; doi:10.1002/ece3.8199)
Supplement: Supplementary file 1 — Fig S1‐S4 [file ECE3-11-16354-s002.docx]

**Appendix 2: Supplementary figures**

No hybrid snowcocks in the Altai – hyper-variable markers can be problematic for phylogenetic inference

Martin Päckert


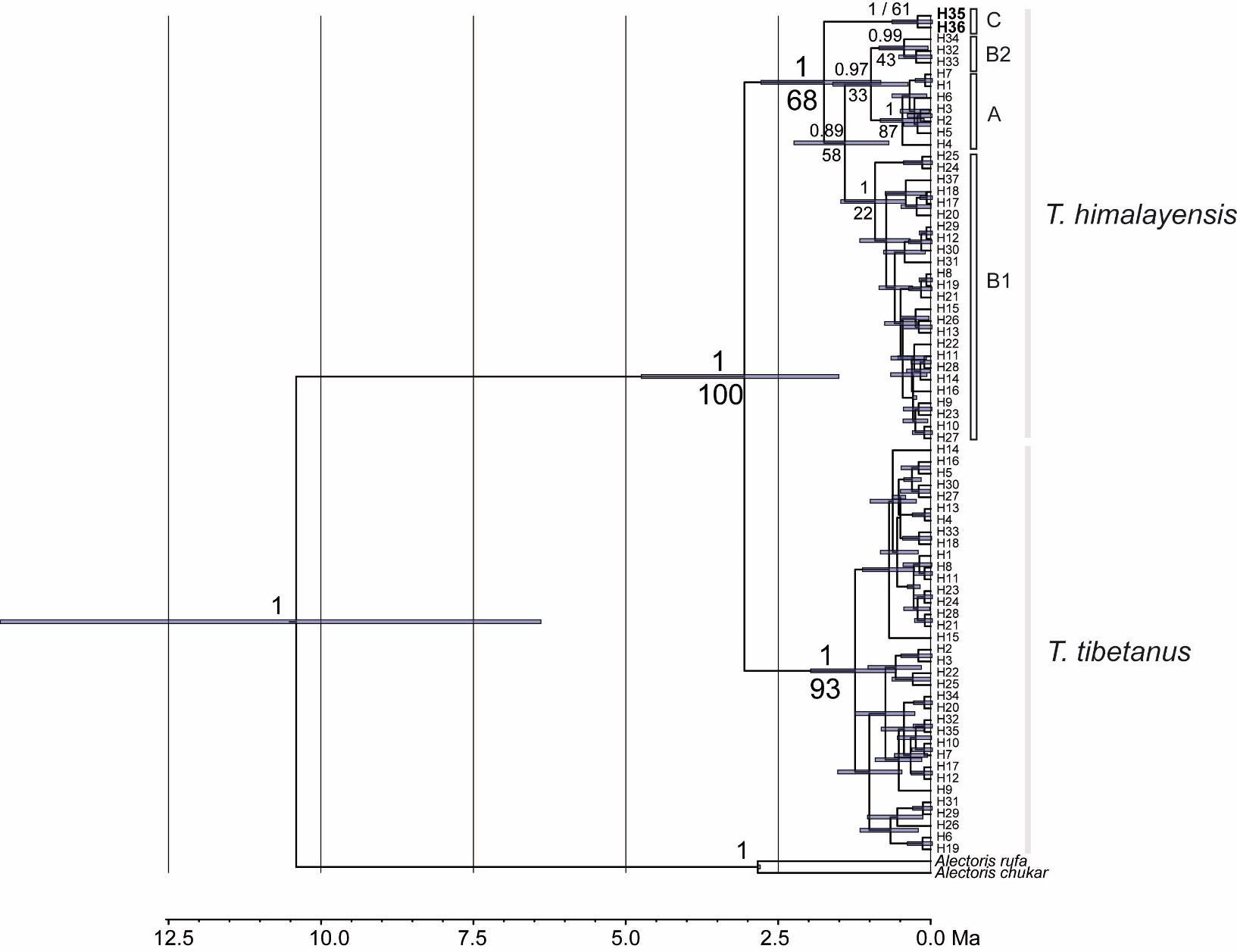


Figure S1: Time-calibrated phylogeny of *Tetraogallus* snowcocks based on the second conservative fragment of the D-loop excluding the hypervariable region (751 bp); Ding et al.’s (2020) original data set (n= 74).


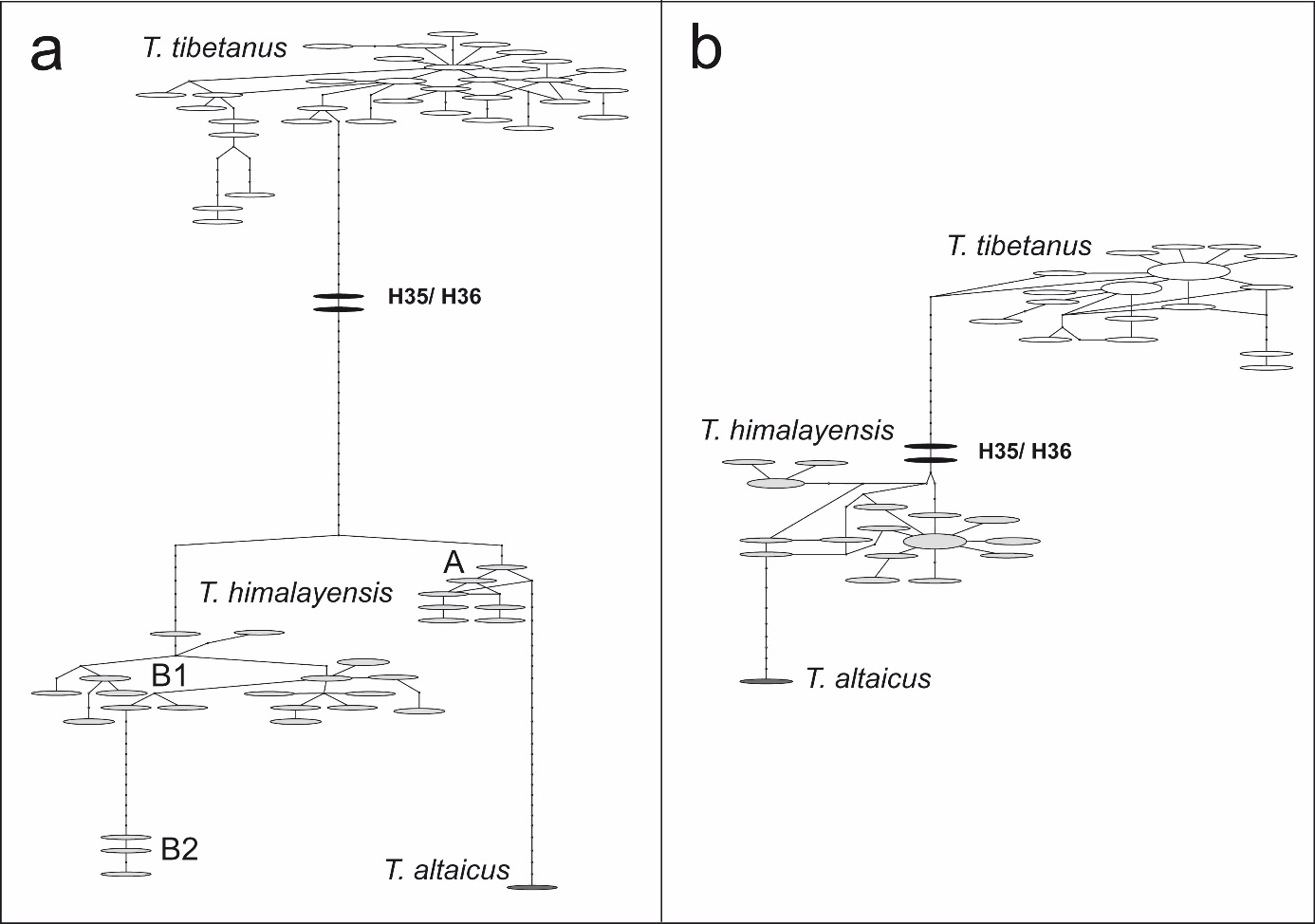


Figure S2: TCS networks for D-loop haplotypes of *Tetraogallus* snowcocks with gaps treated as a 5th character: a) 890 bp manual alignment, b) 751 without the hypervariable region.


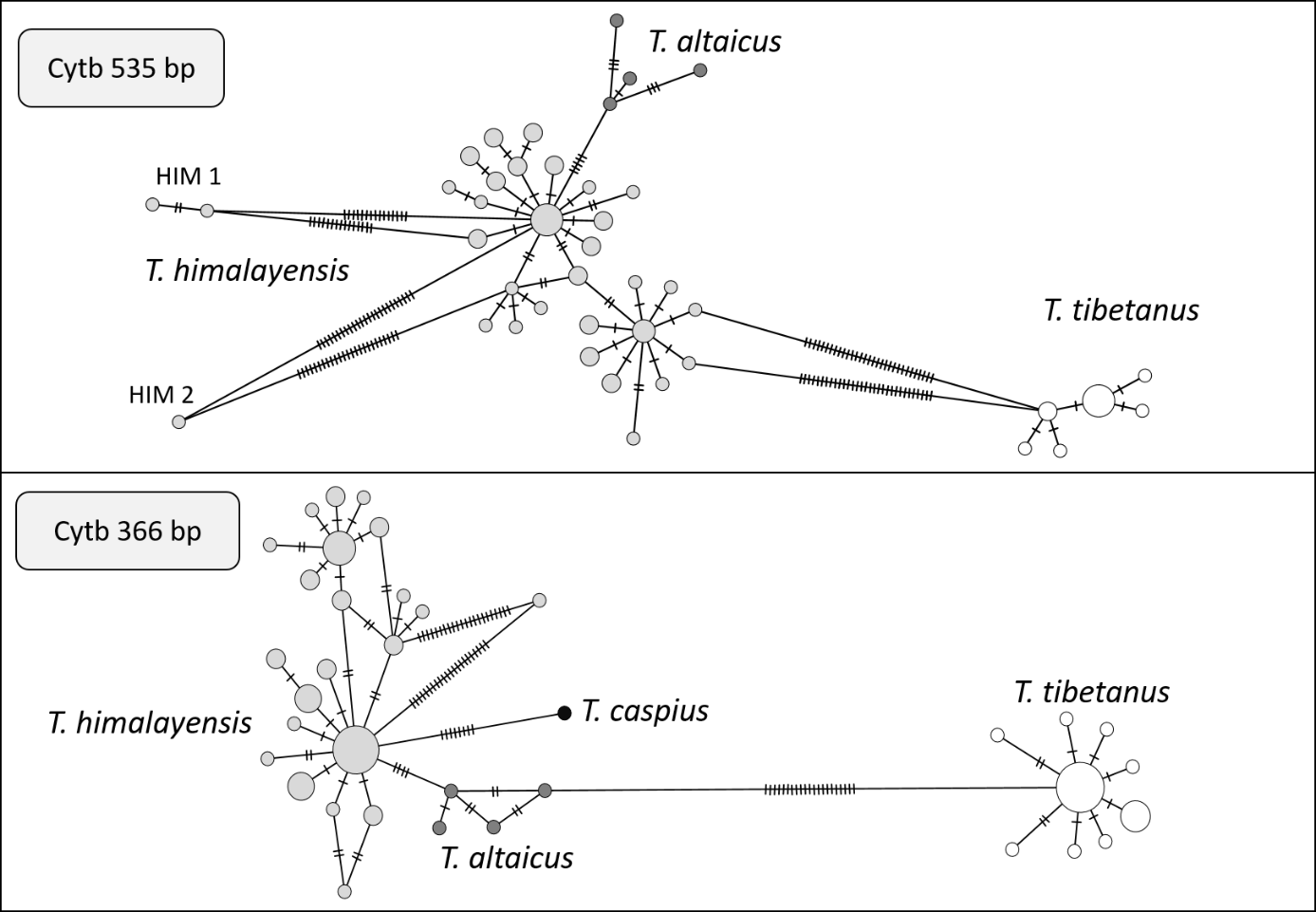


Figure S3: Minimum spanning networks of cytochrome-b based on an alignment of 535 bp (above: cut down to the shortest sequence of *T. altaicus*; excluding the shorter *T. caspicus*) and on an alignment of 366 bp (below: cut down to the shortest sequence of *T. caspicus*).


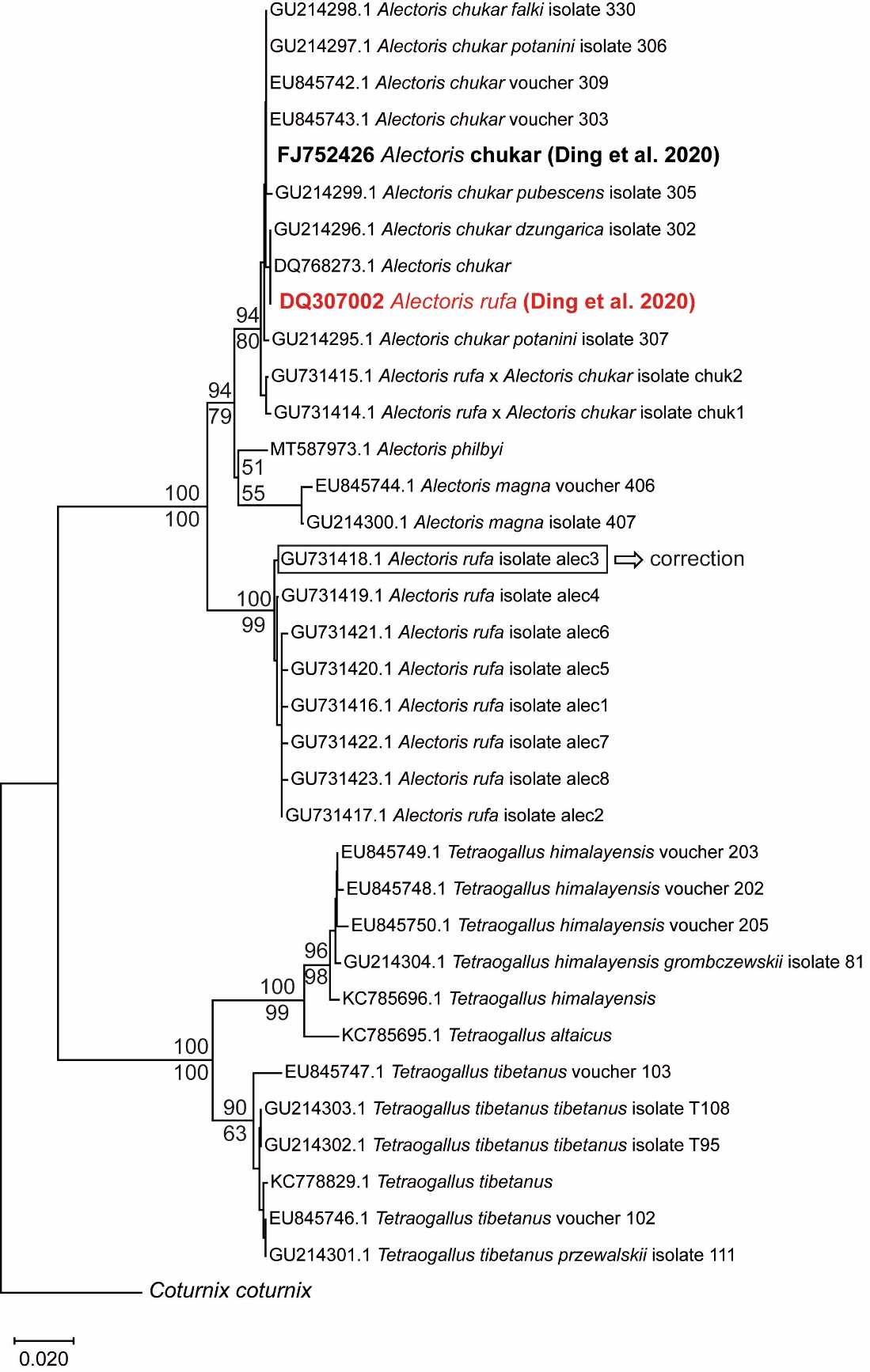


Figure S4: Neighbor-joining tree showing phylogenetic relationships of ND2 sequences of the Chukar partridge (*Alectoris chukar*) and the red-legged partridge (*A. rufa*); marked bold the two sequences used by Ding et al. (2020) for time calibration of their multi-locus tree; the sequence marked in red was chosen by Ding et al. (2020) as being representative for *A. rufa*, however it was apparently inferred from a misidentified specimen; marked in a box the alternative sequence used for inference of divergence time estimates based on a corrected multi-locus data set in this study.
