# Supplementary material for: No hybrid snowcocks in the Altai—Hyper‐variable markers can be problematic for phylogenetic inference
Source: Ecol Evol. 2021 Oct 5;11(22):16354–64. doi: 10.1002/ece3.8199 (PMC8601899; doi:10.1002/ece3.8199)
Supplement: Supplementary file 2 — Table S1‐S4 [file ECE3-11-16354-s001.docx]

**Appendix 1: Supplementary Tables**

No hybrid snowcocks in the Altai – hyper-variable markers can be problematic for phylogenetic inference

Martin Päckert

Table S1: D-loop sequences used for reconstruction of single-locus phylogenies and haplotype networks; haplotypes (hap.) according to numeration in original studies („source“) by An et al. (2015) and Wang et al. (2011).

| **Ding et al. (2020): data set 1= 890 bp, data set 2 = 1163 bp** | | | | | |
| --- | --- | --- | --- | --- | --- |
| **acc no** | **species** | **hap.** | **bp** | **source** | **Ding et al. (2020)** |
| JX136799 | *Tetraogallus tibetanus* | H1 | 884 | An et al. (2015) | yes |
| JX136800 | *Tetraogallus tibetanus* | H2 | 884 | An et al. (2015) | yes |
| JX136801 | *Tetraogallus tibetanus* | H3 | 884 | An et al. (2015) | yes |
| JX136802 | *Tetraogallus tibetanus* | H4 | 883 | An et al. (2015) | yes |
| JX136803 | *Tetraogallus tibetanus* | H5 | 884 | An et al. (2015) | yes |
| JX136804 | *Tetraogallus tibetanus* | H6 | 884 | An et al. (2015) | yes |
| JX136805 | *Tetraogallus tibetanus* | H7 | 884 | An et al. (2015) | yes |
| JX136806 | *Tetraogallus tibetanus* | H8 | 884 | An et al. (2015) | yes |
| JX136807 | *Tetraogallus tibetanus* | H9 | 884 | An et al. (2015) | yes |
| JX136808 | *Tetraogallus tibetanus* | H10 | 884 | An et al. (2015) | yes |
| JX136809 | *Tetraogallus tibetanus* | H11 | 884 | An et al. (2015) | yes |
| JX136810 | *Tetraogallus tibetanus* | H12 | 884 | An et al. (2015) | yes |
| JX136811 | *Tetraogallus tibetanus* | H13 | 884 | An et al. (2015) | yes |
| JX136812 | *Tetraogallus tibetanus* | H14 | 884 | An et al. (2015) | yes |
| JX136813 | *Tetraogallus tibetanus* | H15 | 884 | An et al. (2015) | yes |
| JX136814 | *Tetraogallus tibetanus* | H16 | 884 | An et al. (2015) | yes |
| JX136815 | *Tetraogallus tibetanus* | H17 | 884 | An et al. (2015) | yes |
| JX136816 | *Tetraogallus tibetanus* | H18 | 884 | An et al. (2015) | yes |
| JX136817 | *Tetraogallus tibetanus* | H19 | 884 | An et al. (2015) | yes |
| JX136818 | *Tetraogallus tibetanus* | H20 | 884 | An et al. (2015) | yes |
| JX136819 | *Tetraogallus tibetanus* | H21 | 884 | An et al. (2015) | yes |
| JX136820 | *Tetraogallus tibetanus* | H22 | 884 | An et al. (2015) | yes |
| JX136821 | *Tetraogallus tibetanus* | H23 | 883 | An et al. (2015) | yes |
| JX136822 | *Tetraogallus tibetanus* | H24 | 884 | An et al. (2015) | yes |
| JX136823 | *Tetraogallus tibetanus* | H25 | 884 | An et al. (2015) | yes |
| JX136824 | *Tetraogallus tibetanus* | H26 | 884 | An et al. (2015) | yes |
| JX136825 | *Tetraogallus tibetanus* | H27 | 884 | An et al. (2015) | yes |
| JX136826 | *Tetraogallus tibetanus* | H28 | 884 | An et al. (2015) | yes |
| JX136827 | *Tetraogallus tibetanus* | H29 | 884 | An et al. (2015) | yes |
| JX136828 | *Tetraogallus tibetanus* | H30 | 884 | An et al. (2015) | yes |
| JX136829 | *Tetraogallus tibetanus* | H31 | 884 | An et al. (2015) | yes |
| JX136830 | *Tetraogallus tibetanus* | H32 | 884 | An et al. (2015) | yes |
| JX136831 | *Tetraogallus tibetanus* | H33 | 884 | An et al. (2015) | yes |
| JX136832 | *Tetraogallus tibetanus* | H34 | 884 | An et al. (2015) | yes |
| JX136833 | *Tetraogallus tibetanus* | H35 | 883 | An et al. (2015) | yes |
| GQ343513 | *Tetraogallus himalayensis* | H1 | 1154 | Wang et al. (2011) | yes |
| GQ343514 | *Tetraogallus himalayensis* | H2 | 1154 | Wang et al. (2011) | yes |
| GQ343515 | *Tetraogallus himalayensis* | H3 | 1154 | Wang et al. (2011) | yes |
| GQ343516 | *Tetraogallus himalayensis* | H4 | 1154 | Wang et al. (2011) | yes |
| GQ343517 | *Tetraogallus himalayensis* | H5 | 1154 | Wang et al. (2011) | yes |
| GQ343518 | *Tetraogallus himalayensis* | H6 | 1154 | Wang et al. (2011) | yes |
| GQ343519 | *Tetraogallus himalayensis* | H7 | 1154 | Wang et al. (2011) | yes |
| GQ343520 | *Tetraogallus himalayensis* | H8 | 1154 | Wang et al. (2011) | yes |
| GQ343521 | *Tetraogallus himalayensis* | H9 | 1154 | Wang et al. (2011) | yes |
| GQ343522 | *Tetraogallus himalayensis* | H10 | 1154 | Wang et al. (2011) | yes |
| GQ343523 | *Tetraogallus himalayensis* | H11 | 1154 | Wang et al. (2011) | yes |
| GQ343524 | *Tetraogallus himalayensis* | H12 | 1154 | Wang et al. (2011) | yes |
| GQ343525 | *Tetraogallus himalayensis* | H13 | 1154 | Wang et al. (2011) | yes |
| GQ343526 | *Tetraogallus himalayensis* | H14 | 1154 | Wang et al. (2011) | yes |
| GQ343527 | *Tetraogallus himalayensis* | H15 | 1154 | Wang et al. (2011) | yes |
| GQ343528 | *Tetraogallus himalayensis* | H16 | 1154 | Wang et al. (2011) | yes |
| GQ343529 | *Tetraogallus himalayensis* | H17 | 1154 | Wang et al. (2011) | yes |
| GQ343530 | *Tetraogallus himalayensis* | H18 | 1154 | Wang et al. (2011) | yes |
| GQ343531 | *Tetraogallus himalayensis* | H19 | 1154 | Wang et al. (2011) | yes |
| GQ343532 | *Tetraogallus himalayensis* | H20 | 1154 | Wang et al. (2011) | yes |
| GQ343533 | *Tetraogallus himalayensis* | H21 | 1154 | Wang et al. (2011) | yes |
| GQ343534 | *Tetraogallus himalayensis* | H22 | 1154 | Wang et al. (2011) | yes |
| GQ343535 | *Tetraogallus himalayensis* | H23 | 1154 | Wang et al. (2011) | yes |
| GQ343536 | *Tetraogallus himalayensis* | H24 | 1154 | Wang et al. (2011) | yes |
| GQ343537 | *Tetraogallus himalayensis* | H25 | 1154 | Wang et al. (2011) | yes |
| GQ343538 | *Tetraogallus himalayensis* | H26 | 1154 | Wang et al. (2011) | yes |
| GQ343539 | *Tetraogallus himalayensis* | H27 | 1154 | Wang et al. (2011) | yes |
| GQ343540 | *Tetraogallus himalayensis* | H28 | 1154 | Wang et al. (2011) | yes |
| GQ343541 | *Tetraogallus himalayensis* | H29 | 1154 | Wang et al. (2011) | yes |
| GQ343542 | *Tetraogallus himalayensis* | H30 | 1154 | Wang et al. (2011) | yes |
| GQ343543 | *Tetraogallus himalayensis* | H31 | 1154 | Wang et al. (2011) | yes |
| GQ343544 | *Tetraogallus himalayensis* | H32 | 1154 | Wang et al. (2011) | yes |
| GQ343545 | *Tetraogallus himalayensis* | H33 | 1154 | Wang et al. (2011) | yes |
| GQ343546 | *Tetraogallus himalayensis* | H34 | 1154 | Wang et al. (2011) | yes |
| GQ343547 | *Tetraogallus himalayensis* | H35 | 1154 | Wang et al. (2011) | yes |
| GQ343548 | *Tetraogallus himalayensis* | H36 | 1154 | Wang et al. (2011) | yes |
| GQ343549 | *Tetraogallus himalayensis* | H37 | 1154 | Wang et al. (2011) | yes |
| **outgroup** |  |  |  |  |  |
| FJ752426.1 | *Alectoris chukar* | - |  | mitogenome | yes |
| AJ586226.1 | *Alectoris rufa* | - |  | Barbanera et al. (2005) | Yes |
| **full-length D-loop fragments added to data set 2 (1163 bp); data set 3 (total n= 80)** | | | | | |
| KY766922 | *Tetraogallus himalayensis* | - | 16690 | mitogenome | no |
| KY766921 | *Tetraogallus tibetanus* | - | 16692 | mitogenome | no |
| NC_023939 | *Tetraogallus tibetanus* | - | 16692 | mitogenome | no |
| KF027439 | *Tetraogallus tibetanus* | - | 16692 | mitogenome | no |
| GQ343550 | *Tetraogallus tibetanus* | - | 1176 | Wang et al. (2011) | no |
| GQ343551 | *Tetraogallus tibetanus* | - | 1181 | Wang et al. (2011) | no |
| **Putative hybrid species, Altai snowcock, *T. altaicus*, for comparison** | | | | | |
| MW574391 | *Tetraogallus altaicus* | - | 16692 | mitogenome | no |

Table S2: Cytochrome-*b* sequences used for reconstruction of haplotype networks; „isolate“ according to Genbank information for original studies (under „source“).

| **acc no** | **species** | **isolate** | **source** |
| --- | --- | --- | --- |
| AB200357 | *Tetraogallus altaicus* | clone: cytPCR3 | - |
| AB200356 | *Tetraogallus altaicus* | clone: cytPCR2 | - |
| AB200355 | *Tetraogallus altaicus* | clone: cytPCR1 | - |
| AY563127 | *Tetraogallus altaicus* | A1 | Ruan et al. (2005) |
| EU106676 | *Tetraogallus caspius* | CS1 | Ruan et al. (2010) |
| AY563126 | *Tetraogallus himalayensis* | H17 | Ruan et al. (2005) |
| AY563125 | *Tetraogallus himalayensis* | AY45 | Ruan et al. (2005) |
| AY563124 | *Tetraogallus himalayensis* | TS43 | Ruan et al. (2005) |
| AY563123 | *Tetraogallus himalayensis* | TS39 | Ruan et al. (2005) |
| AY563122 | *Tetraogallus himalayensis* | DD31 | Ruan et al. (2005) |
| AY563121 | *Tetraogallus himalayensis* | DD28 | Ruan et al. (2005) |
| AY563120 | *Tetraogallus himalayensis* | DD23 | Ruan et al. (2005) |
| AY563119 | *Tetraogallus himalayensis* | TS21 | Ruan et al. (2005) |
| AY563118 | *Tetraogallus himalayensis* | TS20 | Ruan et al. (2005) |
| AY563117 | *Tetraogallus himalayensis* | TS19 | Ruan et al. (2005) |
| AY563116 | *Tetraogallus himalayensis* | SB10 | Ruan et al. (2005) |
| AY563114 | *Tetraogallus himalayensis* | SB6 | Ruan et al. (2005) |
| AY563113 | *Tetraogallus himalayensis* | H4 | Ruan et al. (2005) |
| AY563112 | *Tetraogallus himalayensis* | H3 | Ruan et al. (2005) |
| AY563111 | *Tetraogallus himalayensis* | H2 | Ruan et al. (2005) |
| AY563110 | *Tetraogallus himalayensis* | H17 | Ruan et al. (2005) |
| AY678108 | *Tetraogallus himalayensis* |  | Ruan et al. (2005) |
| EU839460 | *Tetraogallus himalayensis* | voucher 205 | Ruan et al. (2010) |
| EU839459 | *Tetraogallus himalayensis* | voucher 202 | Ruan et al. (2010) |
| EU581895 | *Tetraogallus himalayensis* | H26 | Ruan et al. (2010) |
| EU581894 | *Tetraogallus himalayensis* | H27 | Ruan et al. (2010) |
| EU581893 | *Tetraogallus himalayensis* | H28 | Ruan et al. (2010) |
| EU581892 | *Tetraogallus himalayensis* | H29 | Ruan et al. (2010) |
| EU581891 | *Tetraogallus himalayensis* | H30 | Ruan et al. (2010) |
| EU581890 | *Tetraogallus himalayensis* | H31 | Ruan et al. (2010) |
| EU581889 | *Tetraogallus himalayensis* | H32 | Ruan et al. (2010) |
| EU581888 | *Tetraogallus himalayensis* | H33 | Ruan et al. (2010) |
| EU581887 | *Tetraogallus himalayensis* | H34 | Ruan et al. (2010) |
| EU581886 | *Tetraogallus himalayensis* | H35 | Ruan et al. (2010) |
| EU581885 | *Tetraogallus himalayensis* | H36 | Ruan et al. (2010) |
| EU581884 | *Tetraogallus himalayensis* | H37 | Ruan et al. (2010) |
| EU581883 | *Tetraogallus himalayensis* | H38 | Ruan et al. (2010) |
| EU581882 | *Tetraogallus himalayensis* | H39 | Ruan et al. (2010) |
| EU581881 | *Tetraogallus himalayensis* | H40 | Ruan et al. (2010) |
| EU581880 | *Tetraogallus himalayensis* | H41 | Ruan et al. (2010) |
| EU581879 | *Tetraogallus himalayensis* | H42 | Ruan et al. (2010) |
| EU581878 | *Tetraogallus himalayensis* | H43 | Ruan et al. (2010) |
| EU581877 | *Tetraogallus himalayensis* | H44 | Ruan et al. (2010) |
| EU581876 | *Tetraogallus himalayensis* | H45 | Ruan et al. (2010) |
| EU581875 | *Tetraogallus himalayensis* | H46 | Ruan et al. (2010) |
| EU581874 | *Tetraogallus himalayensis* | H47 | Ruan et al. (2010) |
| EU581873 | *Tetraogallus himalayensis* | H48 | Ruan et al. (2010) |
| EU581872 | *Tetraogallus himalayensis* | H49 | Ruan et al. (2010) |
| EU581871 | *Tetraogallus himalayensis* | H50 | Ruan et al. (2010) |
| EU581870 | *Tetraogallus himalayensis* | H51 | Ruan et al. (2010) |
| EU839458 | *Tetraogallus himalayensis* | voucher 201 | Bao et al. (2010) |
| GU214290 | *Tetraogallus himalayensis* | 207 | Bao et al. (2010) |
| GU214289 | *Tetraogallus himalayensis* | 83 | Bao et al. (2010) |
| KY766922 | *Tetraogallus himalayensis* | - | mitogenome |
| NC_027279 | *Tetraogallus himalayensis* | THIM20150414 | mitogenome |
| KY411599 | *Tetraogallus himalayensis* | LSUMZ-B-26339 | mitogenome |
| EU839457 | *Tetraogallus tibetanus* | voucher 101 | Bao et al. (2010) |
| EU839456 | *Tetraogallus tibetanus* | voucher 102 | Bao et al. (2010) |
| GU214288 | *Tetraogallus tibetanus* | 103 | Bao et al. (2010) |
| JX136834 | *Tetraogallus tibetanus* | H01 | An et al. (2015) |
| JX136835 | *Tetraogallus tibetanus* | H02 | An et al. (2015) |
| JX136836 | *Tetraogallus tibetanus* | H03 | An et al. (2015) |
| JX136837 | *Tetraogallus tibetanus* | H04 | An et al. (2015) |
| JX136838 | *Tetraogallus tibetanus* | H05 | An et al. (2015) |
| JX136839 | *Tetraogallus tibetanus* | H06 | An et al. (2015) |
| JX136840 | *Tetraogallus tibetanus* | H07 | An et al. (2015) |
| JX136841 | *Tetraogallus tibetanus* | H08 | An et al. (2015) |
| JX136842 | *Tetraogallus tibetanus* | H09 | An et al. (2015) |
| JX136843 | *Tetraogallus tibetanus* | H10 | An et al. (2015) |
| JX136844 | *Tetraogallus tibetanus* | H11 | An et al. (2015) |
| JX136845 | *Tetraogallus tibetanus* | H12 | An et al. (2015) |
| JX136846 | *Tetraogallus tibetanus* | H13 | An et al. (2015) |
| AY563133 | *Tetraogallus tibetanus* | HX59 | Ruan et al. (2005) |
| AY563132 | *Tetraogallus tibetanus* | HX55 | Ruan et al. (2005) |
| AY563131 | *Tetraogallus tibetanus* | T4 | Ruan et al. (2005) |
| AY563130 | *Tetraogallus tibetanus* | DT20 | Ruan et al. (2005) |
| AY563129 | *Tetraogallus tibetanus* | T1 | Ruan et al. (2005) |
| AY563128 | *Tetraogallus tibetanus* | ZD5 | Ruan et al. (2005) |
| KY766921 | *Tetraogallus tibetanus* | - | mitogenome |
| NC_023939 | *Tetraogallus tibetanus* | - | mitogenome |
| KF027439 | *Tetraogallus tibetanus* | - | mitogenome |

Table S3: Multi-locus sequence data set from Ding et al. (2020) used for control of divergence time estimates with false and corrected ND2 sequence for the outgroup taxon *Alectoris rufa* and *Francolinus swainsonii* added as a further outgroup; Ding et al. (2020) had not included the D-loop sequence MW574391 for the putative hybrid form *T. altaicus*, because it had not been available at that time.

|  | **12S** | **COX1** | **CYTB** | **ND2** | **D-loop** | **CLTC** | **CLTCL1** | **RHO** | **EEF2** |
| --- | --- | --- | --- | --- | --- | --- | --- | --- | --- |
| *T. tibetanus* | NC_023939.1 | NC_023939.1 | NC_023939.1 | NC_023939.1 | NC_023939.1 | KC778959.1 | KC778851.1 | KC778916.1 | KC778873.1 |
| *T. himalayensis* | NC_027279.1 | NC_027279.1 | NC_027279.1 | NC_027279.1 | NC_027279.1 | KC785632.1 | KC785646.1 | KC785713.1 | KC785663.1 |
| *T. caspius* | KJ001802.1 | – | EU106676.1 | – | – | – | – | – | – |
| *T. altaicus* | KC785617.1 | GQ482760.1 | AY563127.1 | KC785695.1 | [MW574391] | KC785631.1 | KC785645.1 | KC785712.1 | KC785662.1 |
| *A. chukar* | FJ752426.1 | FJ752426.1 | FJ752426.1 | FJ752426.1 | FJ752426.1 | KC749574.1 | KC749619.1 | EF569435.1 | KC749686.1 |
| *A. rufa* | KC749448.1 | GU951807.1 | HG940463.1 | DQ307002.1 (wrong) GU731418 (correct) | AJ586226.1 | KC749576.1 | KC749621.1 | EF569436.1 | KC749688.1 |
| *F. swainsonii* | KC785613.1 | – | AM236907.2 | KC785691.1 | DQ834532.1 | KC785628.1 | – | KC785708.1 | KC785658.1 |

Table S4: ND2 sequences used for reconstruction of single-locus phylogeny for control of outgroup sequences of partridges (*Alectoris*); haplotypes according to numeration in original studies („source“) by An et al. (2015) and Wang et al. (2011); sequences used by Ding et al. (2020) for reconstruction of their D-loop tree indicated by „yes“.

| **acc no** | **species** | **isolate** |
| --- | --- | --- |
| FJ752426.1 | *Alectoris chukar* | - |
| EU845743 | *Alectoris chukar* | voucher 303 |
| EU845742 | *Alectoris chukar* | voucher 309 |
| GU214299 | *Alectoris chukar* | 305 |
| GU214298 | *Alectoris chukar* | 330 |
| GU214297 | *Alectoris chukar* | 306 |
| GU214296 | *Alectoris chukar* | 302 |
| GU214295 | *Alectoris chukar* | 307 |
| DQ768273 | *Alectoris chukar* | - |
| EU845744 | *Alectoris magna* | voucher 406 |
| GU214300 | *Alectoris magna* | 407 |
| MT587973 | *Alectoris philbyi* | - |
| GU731423 | *Alectoris rufa* | alec8 |
| GU731422 | *Alectoris rufa* | alec7 |
| GU731421 | *Alectoris rufa* | alec6 |
| GU731420 | *Alectoris rufa* | alec5 |
| GU731419 | *Alectoris rufa* | alec4 |
| GU731418 | *Alectoris rufa* | alec3 |
| GU731417 | *Alectoris rufa* | alec2 |
| GU731416 | *Alectoris rufa* | alec1 |
| DQ307002 | *Alectoris cf. rufa* | - |
| GU731415 | *Alectoris rufa x Alectoris chukar* | chuk2 |
| GU731414 | *Alectoris rufa x Alectoris chukar* | chuk1 |
| KR732853 | *Coturnix coturnix* | - |
| KC785695 | *Tetraogallus altaicus* | - |
| EU845750 | *Tetraogallus himalayensis* | voucher 205 |
| EU845749 | *Tetraogallus himalayensis* | voucher 203 |
| EU845748 | *Tetraogallus himalayensis* | voucher 202 |
| GU214304 | *Tetraogallus himalayensis* | 81 |
| KC785696 | *Tetraogallus himalayensis* | - |
| EU845747 | *Tetraogallus tibetanus* | voucher 103 |
| EU845746 | *Tetraogallus tibetanus* | voucher 102 |
| GU214303 | *Tetraogallus tibetanus* | T108 |
| GU214302 | *Tetraogallus tibetanus* | T95 |
| GU214301 | *Tetraogallus tibetanus* | 111 |
| KC778829 | *Tetraogallus tibetanus* | - |
